# Effects of Adding Butyrate or Exogenous Fibrolytic Enzymes on Ruminal Epithelium, Metabolism, and Microbiota of Beef Cattle

**DOI:** 10.3390/ani15233380

**Published:** 2025-11-21

**Authors:** Daniel Moretto Casali, Kalista Eloisa Loregian, Leandro Aparecido Ferreira da Silva, Ana Laura Januário Lelis, Rodrigo José de Oliveira, Daniel Hideki Mariano Watanabe, Johnny Maciel Souza, Garret Suen, Danilo Domingues Millen

**Affiliations:** 1 School of Agricultural Sciences, São Paulo State University (UNESP), Botucatu 18618-618, SP, Brazil; 2 School of Agricultural and Veterinary Sciences, São Paulo State University (UNESP), Jaboticabal 14884-900, SP, Brazil; 3 Department of Bacteriology, University of Wisconsin-Madison, Madison, WI 53707, USA

**Keywords:** additives, feed additives, metabolism, microbiology, ruminal papillae

## Abstract

Feedlot cattle produce high concentrations of short-chain fatty acids (SCFAs) through ruminal fermentation, with butyrate playing a crucial role in epithelial development and SCFA absorption. This study evaluated the effects of dietary sodium butyrate or exogenous fibrolytic enzymes on the ruminal epithelium and microbiota. Three rumen-cannulated Aberdeen Angus steers were assigned to a replicated 3 × 3 Latin square design, totaling six experimental periods. Each period lasted 28 d, comprising 14 d of adaptation and 14 d of finishing. Diets only differed in additive inclusion: CON—control; ENZ—exogenous enzyme complex (0.01% of the dry matter [DM]); BUT—sodium butyrate (0.3% DM during adaptation; 0.1% during finishing). The ENZ reduced the ruminal temperature (*p* = 0.04), and Diplodinium population (*p* = 0.03). The CON animals had greater lipopolysaccharide-binding protein concentration (*p* = 0.02). No treatment effects were observed for the alpha and beta diversity of bacterial communities, but shifts in abundance at the phylum level were detected for Bacteroidetes, Firmicutes, and Proteobacteria. Papillae dimensions were not significantly altered by treatments (*p* > 0.05). These findings suggest that while BUT and ENZ have some influence on the ruminal environment, their effects at the tested doses were limited, indicating the need for further studies with varying doses or combinations.

## Simple Summary

In modern beef production, cattle are often fed high-grain diets to enhance performance, but this can cause digestive stress if the ruminal papillae are not properly developed. We evaluated whether adding dietary sodium butyrate or exogenous fibrolytic enzymes could improve ruminal epithelial development and metabolism in feedlot cattle. The enzymes showed minor benefits for digestion, but neither additive significantly improved the ruminal papillae structure at the tested doses. These findings indicate that while such additives may have potential, their effects were limited under our conditions, and further research with different doses or combinations is needed. Proper papillae development is essential not only for maintaining rumen health but also for maximizing cattle growth and productivity, since it ensures the efficient absorption of short-chain fatty acids, the main energy source for ruminants.

## 1. Introduction

Ruminal digestion is a complex process involving the interaction between microorganisms, substrates, and metabolites, primarily occurring through degradation and fermentation. Degradation takes place in the extracellular environment of the rumen, where microbial enzymes break down dietary macromolecules into smaller compounds [1]. Fermentation, in contrast, is intracellular and anaerobic, producing energy and releasing metabolites [2,3].

Among the primary fermentation metabolites are short-chain fatty acids (SCFAs), including acetate, propionate, and butyrate, which supply up to 70% of the metabolizable energy of the host. These compounds are absorbed across the ruminal papillae, whose epithelial structure increases the absorptive surface area. Among the SCFAs, butyrate stands out for promoting epithelial development through signaling pathways that regulate cell proliferation, differentiation, metabolism, and nutrient absorption [4].

High-grain diets are commonly used in feedlot systems to enhance animal performance. However, the resulting increase in fermentative activity demands a well-adapted ruminal epithelium to prevent disorders such as ruminal acidosis [5]. Butyrate is considered the primary inducer of epithelial development [6]. However, its relative concentration remains low, typically ranging from 10% to 15% of total SCFAs in fiber- or grain-based diets [7].

In this context, sodium butyrate supplementation has been investigated as a strategy to enhance ruminal epithelial growth. This additive may increase papillae height, mucosal thickness, and the expression of genes associated with cellular proliferation [8,9] while also positively modulating the microbiota and epithelial barrier integrity [10]. Another approach involves the use of fibrolytic enzymes, which improve fiber digestibility and increase SCFA production, including butyrate [1,11]. Although sodium butyrate and fibrolytic enzymes have been individually studied, most investigations have focused either on fermentation parameters or animal performance. There is still limited evidence regarding their specific effects on ruminal epithelial morphology and microbial communities, especially under high-concentrate diets where epithelial adaptation is essential to prevent metabolic disorders. Thus, the integrated evaluation of epithelial development, fermentation, and microbiota remains a knowledge gap in the current literature.

Therefore, this study hypothesizes that dietary supplementation with sodium butyrate or fibrolytic enzymes may modulate ruminal metabolism, thereby promoting ruminal epithelial development and positively influencing animal physiology. The novelty of this work lies in the integrated evaluation of histological, fermentative, microbial, and physiological parameters in beef cattle, providing new insights into the mechanisms through which sodium butyrate and fibrolytic enzymes may enhance ruminal health and efficiency under feedlot conditions. Thus, the objective of this study was to evaluate the effects of sodium butyrate or fibrolytic enzyme supplementation on the ruminal epithelium and microbiota, as well as on fermentative and blood parameters in feedlot Angus cattle.

## 2. Materials and Methods

### 2.1. Animals, Experimental Location, and Ethical Standards

All procedures were approved by the Institutional Animal Care and Use Committee (protocol 08/2019.SP1). The study was conducted at the College of Agricultural and Technological Sciences, São Paulo State University (UNESP), Dracena Campus, São Paulo, Brazil, using the experimental feedlot facility for beef cattle. Three rumen-cannulated, castrated Angus steers (average body weight [BW] ≈ 680 kg; 48 months of age) were used. It is noteworthy to mention that animals were born in the same month, on the same farm, and fed together since then, which minimizes individual animal variation effects.

### 2.2. Experimental Design, Management, Feeding, and Animal Care

The experimental design of the study was a duplicated 3 × 3 Latin square, where each experimental period lasted 28 days. During the pre-experimental period, the animals were kept on pasture, and during the experimental periods, they received diets that differed only in the type of additive offered, following the treatments: CON—Control, without any additive; ENZ—Enzyme Complex (Rovabio^®^ Advance P; Adisseo, São Paulo, Brazil); BUT—Sodium Butyrate (Adimix Easy, Adisseo, São Paulo, Brazil). The additive used in the BUT-fed supplementation (ADIMIX^®^ Easy) contained 68% butyric acid and 32% carrier. At the same time, the enzyme complex (Rovabio^®^ Advance P) consisted of endo-1,4-β-xylanase (at least 25,000 UV/g) and endo-1,3-glucanase (at least 17,200 UV/g) in a 1.45 ratio, both supplied by Adisseo Animal Nutrition (São Paulo, Brazil). At the end of each period, the animals underwent a 7-day washout phase, during which they were released to pasture to standardize the ruminal microbiota and prevent carryover effects from previous treatments.

Experimental diets were formulated using the Large Ruminant Nutrition System (LRNS) software version 1.2.6, level 2, as presented in Table 1. Adaptation was carried out over 14 days following a step-up protocol, in which forage was gradually reduced, and concentrate was increased in the same proportion. The adaptation period consisted of three diets: 5 days on Adaptation Diet 1 (30% forage), 4 days on Adaptation Diet 2 (25% forage), and 5 days on Adaptation Diet 3 (20% forage). After adaptation, animals were fed a finishing diet (15% forage) for 14 days, totaling 28 days for each experimental period.

The inclusion level of the enzyme complex was consistent across the adaptation and finishing diets (0.01% of the dry matter [DM]). In contrast, the inclusion level of sodium butyrate was higher during the adaptation period (0.3% of the DM) compared to the finishing period (0.1% of the DM). Both additives used a mineral supplement as a carrier and were mixed into the diet at the time of feeding directly in the feed bunk. The initial dose of sodium butyrate was established based on the positive outcomes reported by Gorka et al. [12,13] and Guilloteau et al. [14], who observed beneficial effects of sodium butyrate supplementation at a 0.3% inclusion rate in dairy calves. Since these studies focused on the early developmental stage, we adopted the same dosage during the adaptation period of our experiment. Although the physiological stages of young ruminants and adult ruminants undergoing dietary transition differ substantially, the underlying rationale for ruminal epithelium development in both cases stems from the specific challenges each group encounters. In young ruminants, the transition from a pre-ruminant to a functional ruminant state requires rapid and abrupt development of the ruminal epithelium to enable SCFA absorption and support microbial fermentation. In contrast, adult ruminants shifting from a forage-based to a grain-based diet must similarly undergo epithelial proliferation and microbiota adaptation to accommodate increased fermentable carbohydrate intake, enhanced SCFA production, and prevention of epithelial damage from reduced pH. Thus, both scenarios demand structural and functional maturation of the ruminal epithelium, albeit triggered by distinct developmental or dietary stressors. After this phase, the dosage was reduced, assuming that the gastrointestinal epithelium had already adapted to the new diet and no longer required the same level of support.

Cattle were fed ad libitum once daily in the morning (08:00). The amount of diet offered was adjusted based on the refusals from the previous day. Refusals were recorded, collected for chemical analyses, and then discarded.

The pens were partially concrete-floored, with good air circulation, and each pen housed one animal (72 m^2^ and 6 m of linear bunk per animal). Water was always available through an automatic water trough (0.90 × 1.0 × 2.0 m).

### 2.3. Feeding Behavior and Particle Size Selectivity

Feeding behavior and particle size selectivity were assessed on day 16 of each experimental period, according to Robles et al. [15]. Behavioral observations were recorded every 5 min over 24 h, distinguishing between idling, rumination, and feeding activities. In addition to behavioral data, dry matter intake (DMI) was measured, and samples of the offered diet and refusals were collected to determine DM [16] and neutral detergent fiber (NDF) contents, as described by Van Soest [17]. The results were used to calculate meal duration in minutes, DMI per meal, NDF intake rate, and rumination rate [18].

For sorting indices, samples of the offered diet and refusals collected during the behavioral assessment were analyzed using the Penn State Particle Size Separator (PSPS; PennState Extension, State College, PA, USA), as reported by Heinrichs and Kononoff [19]. The sorting index was determined by calculating the “actual intake/expected intake” ratio based on sieve diameter: 19 mm (long), 8 mm (medium), 1.18 mm (short), and the material collected in the pan (fine). Results for this ratio can be equal to 1 (no sorting), less than 1 (sorting against, indicating avoidance), or greater than 1 (preferential selection).

### 2.4. In Situ Ruminal Degradability

The in situ degradability trial was conducted on days 17, 18, and 19 of the experimental periods, following the methodology proposed by Mehres and Ørskov [20]. Nylon bags (50 μm pore size, 10.0 × 19.0 cm) containing approximately 15 g of the experimental diet samples—previously dried in a forced-air oven at 65 °C for 72 h—were used.

The bags were incubated in the rumen of the animals for 0, 6, 12, 24, 48, and 72 h on the days indicated above. For each incubation time, three replicate nylon bags were used. After removal from the rumen, the bags were washed under running water until the wash water ran clear. These were then dried in a forced-air oven at 65 °C for 72 h for the subsequent analysis of starch [21], crude protein (CP) [16], NDF, and acid detergent fiber (ADF) [17], and ash [16].

Degradability data were fitted using the model of Ørskov and McDonald [22], while potential and effective degradability were estimated using models proposed by Ørskov et al. [23]. Potential of rumen degradability was calculated using the following equation:

$$p=a+b\;\times (1-\;e^{-c\times t})$$

where *p* is the percentage of the degraded substrate, *a* is the water-soluble and rapidly degradable fraction of the feed, *b* is the insoluble but potentially degradable fraction of the feed, *c* is the degradation rate of fraction *b*, and *t* is the incubation time. On the other hand, to calculate the degradability considering the passage rate at 2, 5, and 8%, the following equation was used:

$$ED=\;\frac{a+(b\;\times c)}{c+k},$$

where *ED* is the effective degradability considering passage rate, *a*, *b*, and *c* correspond to the same parameters of the aforementioned equation, and *k* is the passage rate of the solid particles in the rumen.

### 2.5. Apparent Total Tract Digestibility of DM and Nutrients

The apparent total tract digestibility of nutrients (DM, CP, NDF, ADF, starch, and ash) was determined using titanium dioxide as an external marker. From days 15 to 24 of each experimental period, 1 g/kg of DM of titanium dioxide was administered via the cannula, half at 08:00 and the other half at 16:00, to ensure a more constant flow of the marker throughout the entire gastrointestinal tract over the course of the day. From days 20 to 24, fecal, diet, and refusal samples were collected. Samples of diet and refusals were used for nutrient analysis and were not subjected to titanium dioxide analysis, as the marker was administered via cannula. Fecal samples were collected twice daily (mid-morning and mid-afternoon) to minimize confounding effects arising from diurnal variations in marker and nutrient excretion. Following the collection period, all fecal samples (eight per animal) were combined into a single composite sample, which was analyzed for titanium dioxide and nutrient concentrations. After collection, samples were ground to pass through a 1 mm screen, and a composite sample representing 5 days of collection was formed. The apparent digestibility coefficients of the nutrients were calculated according to the concentration of titanium dioxide in the fecal samples following the methodology proposed by Titgemeyer et al. [24] as follows:

$$Apparent\;nutrient\;digestibility,\;\%\;=100\times \left(\frac{concentration\;of\;marker\;in\;feed}{concentration\;of\;markerin\;feces}\times \frac{concentration\;of\;nutrient\;in\;feces}{concentration\;of\;nutrient\;in\;feed}\right)$$

### 2.6. Continuous Measurement of Ruminal pH

Values for pH, temperature, and redox potential in the rumen were recorded every 5 min on days 25 and 26 using the “Lethbridge Research Center” (LRCpH) measurement system, developed with a data logger (model T7-1 LRCpH, Dascor, Escondido, CA, USA). Two 900 g weights were attached to the logger to ensure it remained close to the ruminal epithelium, positioned in the ventral region of the rumen [25].

Data were used to calculate the minimum, mean, and maximum pH, as well as the duration (in minutes) that the pH remained below 5.2, 5.6, and 6.2, and the area under the curve (AUC) for pH levels below these thresholds, according to Bevans et al. [26]. Sensor calibration was performed using pH 7.0 and 4.0 solutions to adjust the measured data.

### 2.7. Evaluation of Ruminal Fermentation Products

Samples of ruminal fluid were collected at 0, 4, 8, and 12 h after feeding on day 26 of each experimental period. At each time point, samples were collected from different rumen sites and squeezed through a fine mesh. Approximately 50 mL of ruminal fluid was taken to the laboratory and prepared for the analysis of SCFA, lactate, and ammonia nitrogen (NH_3_-N). The 0 h samples were collected before feeding.

During sample preparation, 25 mL of ruminal fluid was centrifuged at 3500 rpm for 15 min, and aliquots of the supernatant were used for subsequent analyses.

SCFA concentrations were determined using gas chromatography, as described by Erwin et al. [27]. Two milliliters of supernatant was placed in a stoppered test tube with 0.4 mL of formic acid and stored at −20 °C until analysis using a gas chromatograph (Finnigan, model 9001, Thermo Fisher Scientific, Waltham, MA, USA) equipped with a Megabore column (OV-351, 1 micron, 30 m long, 0.53 mm internal diameter, Ohio Valley, Marietta, OH, USA).

Ammonia nitrogen concentration was determined by placing 2 mL of the supernatant in test tubes containing 1 mL of 1N sulfuric acid and storing at −20 °C until analysis, which followed the colorimetric method described by Kulasek [28] and adapted by Foldager [29]. Lactate concentration in ruminal fluid was determined using a colorimetric method adapted from Pryce [30]. Briefly, 0.05 mL of ruminal fluid was mixed with 3.95 mL of precipitating reagent (10 g sodium tungstate, 22 mL of 90% orthophosphoric acid, and 5 g copper sulfate per liter). After centrifugation (5 min, 2000 rpm), 1 mL of the supernatant was transferred to Pyrex tubes, and 6 mL of sulfuric acid was added rapidly. After cooling, 0.1 mL of p-hydroxybiphenyl solution in dimethylformamide was added, and the mixture was incubated in a boiling water bath for 90 s. The absorbance was measured at 565 nm. The concentration of lactic acid was calculated by proportionality using a lithium lactate standard (40 mg/100 mL).

### 2.8. Sequencing of Ruminal Bacterial Communities

On day 26 of each experimental period, 4 h after feeding, 50 mL of ruminal content (solid and liquid fractions) was collected in sterile tubes and frozen at −80 °C. After thawing, 10 mL of the contents was placed in a stomacher bag and 40 mL of DNA extraction buffer was added. A subsample of the recombined contents underwent DNA extraction using the bead-beating method described by Weimer et al. [31], which includes mechanical disruption using glass beads, followed by extraction with phenol:chloroform:isoamyl alcohol. The DNA was resuspended in 10 mM Tris HCl with 1 mM EDTA (pH 8.0), quantified fluorometrically using a Qubit (Invitrogen, Carlsbad, CA, USA), and stored at −80 °C prior to library preparation. Samples were diluted to 10 ng/μL to ensure a minimum of 50 ng per PCR reaction.

PCR amplification targeted the variable 4 (V4) region of the bacterial 16S ribosomal RNA (rRNA) gene using universal primers. Universal primers amplifying the Variable 4 region of the bacterial 16S rRNA gene were used to perform polymerase chain reaction (PCR; F-GTGCCAGCMGCCGCGGTAA; R-GACTACHVGGGTWTCTAAT) as described by Kozich et al. [32]. The primers also included unique barcodes for multiplexing and adapters suitable for sequencing using Illumina technology (F-TGATACGGCGACCACCGAGATCTACAC; R-CAAGCAGAAGACGGCATACGAGAT). Each reaction contained 50 ng of DNA, 0.4 μM of each primer, 12.5 μL of 2X Hot Start Ready Mix (KAPA Biosystems, Wilmington, MA, USA), and DNA/RNA-free water to a final volume of 25 μL. Cycling conditions were: initial denaturation at 95 °C for 3 min, followed by 25 cycles of 95 °C for 30 s, 55 °C for 30 s, and 72 °C for 30 s, and a final extension at 72 °C for 5 min.

Amplified products (25 μL) were loaded onto a low-melt agarose gel with five μL of 6X Orange loading dye. Samples displaying bright bands around 380 bp were excised for extraction and subsequent sequencing on an Illumina MiSeq platform (San Diego, CA, USA) using 5% PhiX as a control and MiSeq v4 kits (Illumina, San Diego, CA, USA; 81).

The PCR and sequencing were performed at the Department of Bacteriology, University of Wisconsin, Madison. Sequences were processed with Mothur v.1.40.0 (www.mothur.org/wiki, accessed on 22 March 2025) and aligned against the SILVA 16S rRNA database (v132; 81). Alpha diversity (Chao richness estimator and Shannon diversity index) and beta diversity (Bray–Curtis and Jaccard dissimilarity) were calculated. Additionally, relative abundances of the most abundant and key bacterial communities were determined [31].

### 2.9. Ruminal Ciliate Protozoa

For differential counting of ciliate protozoa, 10 mL of ruminal content (same as for bacterial analysis) was collected 4 h post-feeding on day 26 and stored in tubes with 20 mL of 50% (*v*/*v*) formaldehyde. One milliliter of the diluted sample was stained with brilliant green and glycerol and diluted 30-fold. Counts were performed using a Sedgewick Rafter counting chamber (50 mm × 20 mm × 1 mm, 1 mL capacity, Thermo Fisher Scientific, Waltham, MA, USA) according to Dehority [33], differentiating genera Isotricha, Dasytricha, Entodinium, and Diplodinium over 100 optical fields.

### 2.10. Ruminal Digesta Dynamics

On days 27 (11:00, 3 h post-feeding) and 28 (08:00, pre-feeding), ruminal contents were completely emptied, separated into liquid and solid fractions, and weighed [34]. A 600 g sample was reconstituted in a container replicating the original solid-to-liquid ratio and frozen (−20 °C) for later DM analysis [16]. Based on the solid and liquid masses and DMI, ruminal disappearance rates were expressed in %/h and kg/h.

### 2.11. Metabolic Hormones and Metabolites

On days 1 and 28 of each experimental period, blood samples were collected from the coccygeal vein in heparinized tubes and immediately centrifuged. Plasma was stored at −80 °C and analyzed for haptoglobin, ceruloplasmin, serum amyloid A, immunoglobulins A and G, C-reactive protein, lipopolysaccharide-binding protein (LBP), non-esterified fatty acids (NEFAs), glucose, and insulin [35,36,37,38].

### 2.12. Histology of Ruminal Papillae

Ruminal papillae samples were collected on day 28 of the experimental period. Samples were processed through a series of solutions (1 h each): 70% ethanol, 90% ethanol, 100% ethanol, absolute ethanol I and II, xylene I and II, and paraffin I and II (Histosec^®^, Merck Millipore Brasil, Barueri, São Paulo, Brazil). After these steps, samples were embedded in aluminum blocks with paraffin III (Histosec^®^) and sectioned at six μm on a microtome, followed by Hematoxylin–Eosin and Harris’ Hematoxylin staining [39].

Measurements of papillae height, width, area, keratinized epithelium thickness, and mitotic index were conducted after slide preparation using the Leica Qwin Image Analyzer, Micro capture v.6.17 coupled to a Leica light microscope. Four papillae were randomly selected, and for each papillae, height and area were measured once. Width and keratinized epithelium thickness were measured at four randomly selected points to increase the representativeness of the measurements.

### 2.13. Statistical Analysis

A post hoc power analysis was performed using the GLMPOWER procedure of SAS v9.4 (SAS Institute Inc., Cary, NC, USA), based on the residual variance of key fermentation variables. The duplicated 3 × 3 Latin square design with 3 cannulated animals was chosen because it provided adequate statistical power.

The statistical model included treatment, time (for repeated measures) when appropriate, and their interaction. Treatment was considered as a fixed effect, and time, treatment × time, period, and animal (within square) were considered random effects. For variables related to ruminal short-chain fatty acids, ammonia, and lactate, measured at multiple time points, repeated measures analysis was performed using the animal as the experimental unit. The covariance structures tested were AR(1), ARH(1), ANTE(1), CS, CSH, TOEP, TOEPH, HF, UN, UNR, and VC, and the one selected for each variable was based on the lowest Akaike information criterion (AIC).

Residual normality and variance heterogeneity were tested before proceeding with variance analysis. Data transformations were performed when necessary, using either logarithmic transformation [log(X + 1)] or square root transformation [√(X + 1/2)], depending on the distribution of each variable. Period and animal effects were also considered random effects. Data were analyzed using the PROC MIXED procedure of SAS v9.4 (Cary, NC, USA). Statistical significance was declared at *p* ≤ 0.05, and trends were considered at 0.05 < *p* ≤ 0.10.

Bacterial sequences were grouped into operational taxonomic units (OTUs) at 97% sequence similarity. Good’s coverage [40] was calculated in Mothur v1.48.4 for all samples, and a Good’s coverage ≥0.95 was considered sufficient to ensure sequencing depth. The OTU counts were normalized to 10,000 sequences per sample before analysis.

Alpha diversity (within-sample diversity) was assessed using Chao1 richness [41] and Shannon diversity index [42]. Differences in diversity between treatments were analyzed using two-way ANOVA in R v3.2.1 [43]. Data on ruminal bacterial communities were tested for normality (Shapiro–Wilk) before performing the analysis. Beta diversity (between-sample composition) was assessed using Bray–Curtis dissimilarity and visualized by non-metric multidimensional scaling (NMDS). Community structure differences were tested using permutational multivariate analysis of variance (PERMANOVA) with the vegan package in R v2.5-2 [44], pairwise comparisons were false discovery rate (FDR-corrected).

## 3. Results

No statistical differences were observed for DMI during the adaptation (*p* = 0.82) and finishing phases (*p* = 0.78) or as a percentage of body weight (*p* = 0.78; Table 2). Treatments did not affect idling (*p* = 0.30), rumination (*p* = 0.12), or feeding activities (*p* = 0.53), nor the selectivity for medium (*p* = 0.61), short (*p* = 0.18), or fine particles (*p* = 0.16); Table 3). However, the sorting index for long particles decreased for animals in the ENZ treatment compared to the other treatments (*p* < 0.01; Table 3). Treatment tended to affect DMI per meal (*p* = 0.06), whereas ENZ supplementation tended to reduce DMI per meal relative to CON, and BUT did not differ from either of the treatments (Table 3).

No treatment effects were observed on in situ ruminal degradability parameters of DM (*p* ≥ 0.33), NDF (*p* ≥ 0.31), ADF (*p* ≥ 0.74), and CP (*p* ≥ 0.14). However, the BUT treatment tended to have greater starch degradability compared to the other treatments (*p* = 0.09; Table 4). The apparent digestibility of DM (*p* = 0.69), NDF (*p* = 0.14), ADF (*p* = 0.92), CP (*p* = 0.94), and starch (*p* = 0.83), as well as fecal starch concentration, were not affected by treatment (*p* = 0.51; Table 5).

Minimum (*p* = 0.23), mean (*p* = 0.69), and maximum (*p* = 0.38) ruminal pH values were not affected by treatments (Table 6), nor were parameters related to the duration and area under the curve for pH thresholds of 6.2 (*p* = 0.45; *p* = 0.88), 5.6 (*p* = 0.92; *p* = 0.55), and 5.2 (*p* = 0.51; *p* = 0.68). A decreased ruminal temperature (°C) was observed in animals fed ENZ compared to the BUT and CON treatments (*p* = 0.04; Table 6).

The SCFA profile was not influenced by treatments or sampling times (*p* ≥ 0.23; Table 7). Ammonia-N and lactate concentrations did not differ significantly among treatments, although significant variations were observed across sampling times (*p* < 0.01 and *p* = 0.04, respectively).

Populations of Dasytricha (*p* = 0.24) and Entodinium (*p* = 0.15) protozoa did not differ among treatments (Table 8), while decreased Diplodinium counts were observed in ENZ animals, both in absolute values (*p* < 0.01; Table 8) and as a percentage (*p* = 0.03; Table 8). Although Isotricha populations did not differ in absolute values (*p* = 0.12), their percentage decreased for both the ENZ and BUT animals relative to CON (*p* = 0.02; Table 8).

A greater ruminal digesta disappearance rate (%/h) was observed in the ENZ animals compared to the CON and BUT groups (*p* = 0.05). When expressed as kg/h, a similar trend was detected (*p* = 0.09). No significant differences were observed for the other ruminal digesta dynamics parameters (*p* ≥ 0.09; Table 9).

Plasma LBP concentration was greater in the CON treatment compared to ENZ, while BUT did not differ from either (*p* = 0.02; Table 10). A tendency toward greater C-reactive protein (mg/L) levels was observed in the butyrate group (*p* = 0.09), while no significant differences were found for the other inflammation-related proteins. The insulin (*p* = 0.18), NEFA (*p* = 0.99), and glucose (*p* = 0.72) concentrations also did not differ among treatments (Table 10). Plasma lipopolysaccharide-binding protein concentration was greater in the CON treatment compared to ENZ, while BUT did not differ from either (*p* = 0.02; Table 10). A tendency toward greater C-reactive protein (mg/L) levels was observed in the butyrate group (*p* = 0.09; Table 10).

Histological measurements are presented in Table 11, where no treatment effects were observed for papillae dimensions (height [*p* = 0.73], width [*p* = 0.21], and area [*p* = 0.44]), nor microscopic parameters such as keratin layer thickness and mitotic index (*p* ≥ 0.23).

**Figure 1:**
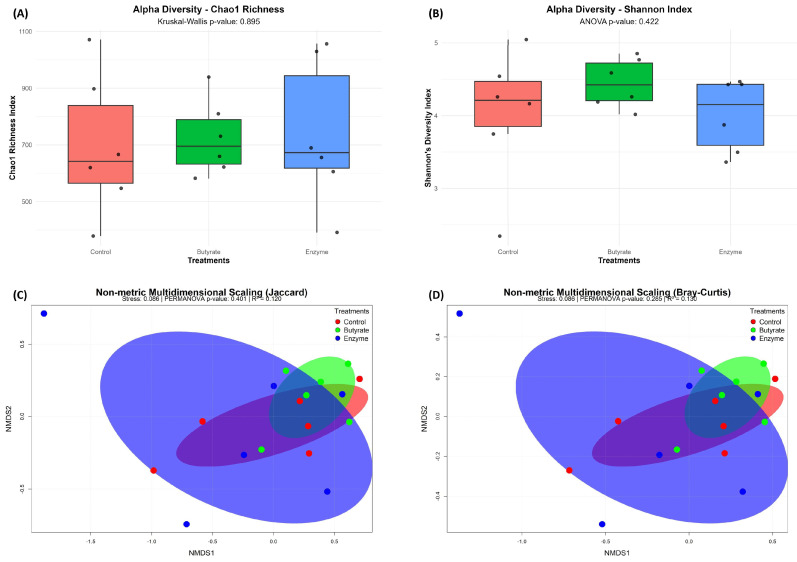
Alpha and beta diversity of the ruminal microbiota in ruminally cannulated feedlot Angus steers supplemented with fibrolytic enzymes or sodium butyrate. The analysis compared three treatment groups: Control, Butyrate, and Enzyme. (**A**) Alpha diversity measured by the Chao1 richness index (Kruskal–Wallis, *p* = 0.895). (**B**) Alpha diversity measured by the Shannon diversity index (ANOVA, *p* = 0.422). For both boxplots, the central line indicates the median, the box limits represent the upper and lower quartiles, and the whiskers extend to 1.5 times the interquartile range. (**C**) Beta diversity visualized through Non-metric Multidimensional Scaling (NMDS) using the Jaccard index (PERMANOVA, *p* = 0.401). (**D**) Beta diversity visualized through NMDS using the Bray–Curtis dissimilarity index (PERMANOVA, *p* = 0.283). In the NMDS plots, each point represents the microbial community of an individual animal, and the ellipses represent the 95% confidence interval for each treatment group.

**Figure 2:**
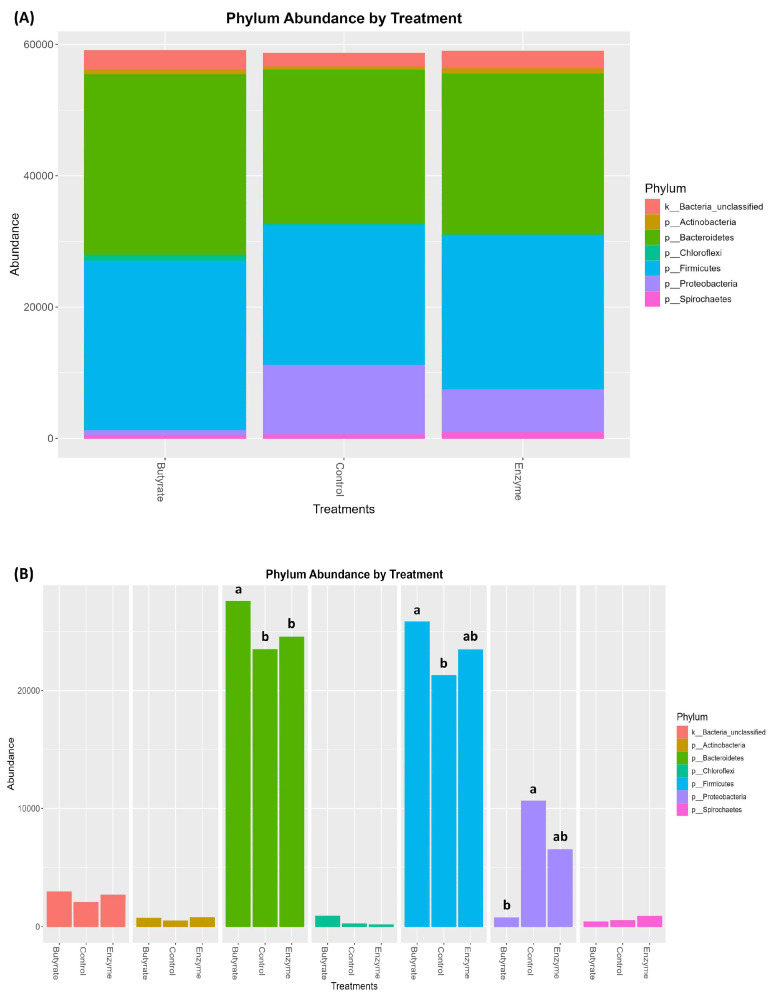
Taxonomic composition of the ruminal bacterial community in Angus steers supplemented with fibrolytic enzymes or sodium butyrate. The analysis shows the abundance of bacterial phyla across the Control, Butyrate, and Enzyme treatment groups. (**A**) Relative abundance of the most prevalent bacterial phyla, illustrating the overall community structure for each treatment. (**B**) Comparison of the abundance of specific bacterial phyla among treatments. Bars with different letters (a, b) indicate a significant difference between treatment groups for that phylum (*p* < 0.05).

No significant differences were observed in the alpha diversity of the ruminal bacterial population with the inclusion of feed additives, as indicated by the Chao (*p* = 0.89; Figure 1A) and Shannon (*p* = 0.42; Figure 1B) indices. Similarly, no treatment effects were detected for beta-diversity indices, including Jaccard (*p* = 0.40; Figure 1C) and Bray–Curtis (*p* = 0.28; Figure 1D). The results presented in Figure 2A,B provide a descriptive overview of the effects of different treatments on the seven most abundant rumen bacterial phyla identified. The Bacteroidetes phylum, followed by Firmicutes and Proteobacteria, emerged as the most prevalent in this study.

In addition, in Figure 2B, significant differences were observed in the relative abundance of major bacterial phyla among treatments. Bacteroidetes were more abundant in the BUT compared with the CON and ENZ groups (*p* < 0.05). Firmicutes showed greater abundance in the BUT group than in the CON group, while the ENZ group presented intermediate values (*p* < 0.05). Conversely, Proteobacteria were significantly more abundant in the CON group than in the BUT group, with the Enzyme group again showing intermediate levels (*p* < 0.05).

## 4. Discussion

The enzyme complex supplementation—comprising xylanases and glucanase in a 1.45:1 ratio was intended to enhance the degradation of cellulose and hemicellulose in forage components, as well as promote the breakdown of xylans present in cereal grains. This ratio approximates the optimal range identified by Eun et al. [45], who reported increased forage degradability in vitro with cellulase:xylanase ratios between 1.16:10 and 1:10. Nevertheless, subsequent research suggests that lower ratios, ranging from 1:2 to 1:4, may be more effective, which are below the level used in the present study and may partly explain the lack of pronounced effects on ruminal fermentation and animal performance. Additionally, butyric acid was supplemented at 38 g/day (0.3% of DM) during the adaptation phase and reduced to 14 g/day (0.1% of DM) in the finishing phase. These inclusion levels did not affect the dry matter intake (DMI), in agreement with the findings from Kowalski et al. [46] and Izumi et al. [47], who also observed no changes in DMI with butyrate doses up to 1.1% of DM across different cattle types. Notably, more substantial physiological effects have only been observed when butyric acid was infused at much higher levels (1.5–6% of DM), as reported by Shen et al. [48], Malhi et al. [49], and Wiese et al. [50].

A higher dose of butyric acid was used during the adaptation phase when greater epithelial development is required to cope with the transition to high-concentrate diets. In the finishing phase, a lower dose was maintained, as the ruminal epithelium was supposed to be adapted. This approach aligns with the functional role of butyrate in promoting epithelial growth and enhancing barrier function [6,13]. As previously noted, studies by Górka et al. [12,13] and Guilloteau et al. [14] demonstrated enhanced ruminal epithelium development in dairy calves supplemented with sodium butyrate. These findings served as the basis for employing higher butyrate doses during the adaptation phase in the present study. However, research is scarce in the current literature evaluating the effects of butyrate supplementation following a dietary transition in cattle. In an effort to elevate butyrate concentrations within the gastrointestinal tract of beef heifers, Watanabe et al. [51] administered butyrate precursors but observed no significant improvements in animal performance.

Studies show that when the epithelium absorbs butyric acid, it is almost completely converted to β-hydroxybutyrate, a ketone body used as an energy source by various tissues once metabolized to acetyl-CoA. Given this energetic use, it is believed that an increase in β-hydroxybutyrate in the blood leads to negative feedback on blood glucose [52,53,54]. However, as reported by Kowalski [52] and Izumi et al. [47], when butyrate doses are insufficient to elevate plasma β-hydroxybutyrate concentrations, parameters such as glucose, NEFA, and insulin are unaffected. Neogrády et al. [55] found a linear insulin response to butyrate infusion only at high doses, supporting the idea that the dosage in the present study was insufficient to promote metabolic changes. While the conversion of butyrate into ketone bodies within ruminal epithelial cells serves as a direct energy source supporting epithelial development, its role extends beyond this metabolic function. Butyrate also exerts indirect effects by stimulating the release of hormones such as insulin-like growth factor-1 (IGF-1) and glucagon-like peptide-2 (GLP-2); IGF-1 is known to promote the proliferation of ruminal epithelial cells [56], while GLP-2 plays a key role in regulating the development and function of the gastrointestinal tract [57]. However, the effects of butyrate appear to be more pronounced when it is released post-ruminally. This has been demonstrated in studies by Górka et al. [13] and Guilloteau et al. [14], who administered sodium butyrate in the milk replacer of dairy calves, and by Watanabe et al. [58], who introduced butyrate precursors into the abomasum of beef heifers. However, butyrate administration did not increase the butyrate proportion in ruminal fluid. The same pattern was observed by Watanabe et al. [58], who reported no increase in butyrate concentration at the site of release (abomasum). We interpret this absence of change in butyrate proportion as potentially related to rapid absorption by the gastrointestinal epithelium, the low dosage employed in both studies, and factors associated with the timing of feeding/infusion relative to sample collection.

Kowalski et al. [46] reported an increase in total SCFA production without changes in ruminal pH, ammonia, or acid proportions. Conversely, Herrick et al. [52], using higher doses of butyrate (1 and 2 g/kg BW), observed a reduction in ruminal N concentration and an increase in pH, possibly due to greater N absorption and increased ruminal papillae surface area.

Ammonia-N concentrations increased 4 h after feeding in a quadratic response (0 h: 4.01, 4 h: 4.51, 8 h: 3.12, 12 h: 3.38 mg/dL), consistent with the peak of fermentation when a greater degradation of protein sources provided high NH_3_ availability to the microbiota. This was corroborated by reduced concentrations at 8- and 12 h post-feeding when these N sources had been depleted [59]. However, no interaction with the treatments was detected, leading to the inference that neither BUT nor ENZ may interfere with ruminal ammonia metabolism. Lactate concentrations peaked at 4 h post-feeding and decreased thereafter in the present study a pattern that coincided with the lowest ruminal pH recorded during the same time point. This temporal dynamic likely reflects a peak in fermentative activity shortly after feeding. Notably, neither sodium butyrate (BUT) nor exogenous fibrolytic enzyme (ENZ) supplementation altered the ruminal lactate concentrations or pH patterns compared to the control at different time points, suggesting that the additives did not affect the fermentation pathways related to lactate metabolism. Although specific lactate-utilizing bacteria such as Selenomonas ruminantium and Megasphaera elsdenii were not identified in this study at a relative abundance greater than 1%, prior research indicates that their activity is reduced at decreased pH levels, which may partially explain the transient lactate accumulation observed [60,61]. Therefore, the results suggest that while fermentation peaked at 4 h post-feeding, the treatments did not significantly modulate the microbial lactate dynamics under the studied conditions.

There was no additive effect on the alpha and beta diversity of the bacterial community. However, the abundance of the Proteobacteria phylum was suppressed with sodium butyrate supplementation, which in turn increased the abundance of phylum Bacteroidetes and Firmicutes. Many butyrate-producing bacteria belong to the Firmicutes phylum [62], and the fact that exogenous butyrate has been added to the rumen, suggests it may have increased the abundance of the Firmicutes phylum. However, it deserves further investigation, since the abundance of Firmicutes is decreased in high-concentrate diets. Additionally, due to the high energy content of the diets, a high prevalence of Bacteroidetes was expected, but feeding butyrate may also have stimulated the growth of bacteria in the Bacteroidetes phylum, especially starch-utilizing bacteria, since the addition of butyrate to the diet increased the potential ruminal degradability of starch. It is worth mentioning that the phylum Proteobacteria is responsible for protein degradation in the rumen, although we did not verify this effect regarding ruminal N concentration [63,64], as described earlier. Therefore, the negative effect of BUT on Proteobacteria may be related to true protein degradation, but this also deserves further investigation.

Although no significant changes were observed in the alpha or beta diversity indices, the shifts in phylum-level composition suggest a functional reorganization of the ruminal microbiome rather than a change in overall community structure or evenness. The concurrent increase in Bacteroidetes and Firmicutes, coupled with the suppression of Proteobacteria in the butyrate-supplemented group, may reflect a microbial community shift toward greater carbohydrate fermentation efficiency and improved ruminal stability. This interpretation is supported by the observed tendency for increased potential starch degradability in the BUT group, which aligns with the known role of Bacteroidetes in complex carbohydrate metabolism. Furthermore, the reduction in Proteobacteria—a phylum often associated with ruminal dysbiosis and inflammatory responses when overabundant—may contribute to a more stable ruminal environment. This is indirectly supported by the reduced plasma LBP concentration observed in the ENZ group, suggesting reduced microbial translocation or systemic inflammation. The lack of changes in overall diversity suggests that the additives modulated specific functional groups without disrupting the broader microbial ecosystem, which may be advantageous for maintaining ruminal homeostasis. These findings highlight the importance of examining compositional changes at the phylum level, even in the absence of shifts in diversity metrics, as they may reveal functionally relevant microbial responses.

The ENZ treatment increased the ruminal DM disappearance rate in terms of %/h and kg/d, without raising SCFA production or animal performance. This suggests that the degraded substrate and fermentation metabolites may have bypassed ruminal utilization by escaping through the reticulo-omasal orifice—similar to results reported by Pinos-Rodrigues et al. [65], who found no effects of xylanase- and glucanase-based enzyme complexes on animal performance or SCFA production but did find enhanced DM and NDF disappearance, suggesting a shift of digestion from the rumen to the intestine.

Similarly, Peters et al. [66] found no differences in animal behavior (rumination), even though ruminal DM disappearance was favored. This maintenance of rumination may help stabilize the ruminal environment, supported by the buffering capacity of saliva. These results align with those of Rice et al. [67] and others, who found no DMI, SCFA profile, or blood β-hydroxybutyrate changes with exogenous fibrolytic enzyme supplementation. Krause et al. [68] concluded that the main factor altering ruminal pH is DMI itself, consistent with the current findings.

Animals in the ENZ treatment showed a reduced sorting index for long particles, the main stimulators of rumination and primary sources of dietary NDF and ADF, suggesting a more stable ruminal environment. This is supported by the stable pH and reduced ruminal temperature observed in ENZ compared to the other treatments, which may have led to a decreased concentration of LBP in the bloodstream of ENZ-fed cattle. Similarly, the greater disappearance rate observed in ENZ-fed cattle may have contributed to the decrease in rumen temperature. Likewise, the greater disappearance rate presented by ENZ-fed cattle may have influenced the reduction in rumen temperature. Additionally, based on these observations, we can infer that ruminal fermentation was milder in the ENZ-supplemented cattle. This reduced fermentative intensity likely preserved a more intact ruminal epithelium and maintained barrier function. Direct measurements of epithelial integrity were not performed in this study. Similar effects have been reported by Beauchemin et al. [11], who observed that exogenous fibrolytic enzymes improved fiber degradation and altered fermentation dynamics in a way that reduced the intensity of ruminal heat production. Additionally, Yang et al. [69] reported that enzyme supplementation can affect feeding behavior and reduce diurnal variation in ruminal parameters. It is noteworthy to mention that no clear associations with microbial shifts could be established within the scope of this study, which may be due to the limited number of animals and the inherent variability of microbiome data. These findings support our interpretation that reduced sorting for long particles. The presence of exogenous enzymes may contribute to a more stable and less exothermic ruminal environment.

The population of Diplodinium protozoa was reduced in animals fed ENZ, likely due to their reliance on fibrous fractions as their main energy source [70]. Thus, exogenous enzymatic action on forages reduced the energy supply for Diplodinium, negatively affecting their populations both numerically and as a percentage. Isotricha protozoa populations were reduced in both the ENZ and BUT treatments compared to CON; these protozoa are sensitive to pH variations, and although no statistical differences were found for the minimum, mean, and maximum pH, CON had the highest values, favoring Isotricha, albeit only as a percentage. Furthermore, the great disappearance rate observed in cattle consuming ENZ compared to those in the control treatment may have contributed to reducing the number of protozoa examined in this study due to their low multiplication rate [33].

The addition of BUT or ENZ did not influence the width or length of ruminal papillae as they did not induce changes in the ruminal SCFA profile. Other studies such as Kowalski et al. [46] and Liu et al. [71] have shown that butyrate supplementation promotes papillae development, potentially increasing the papillary surface area by up to 50%, however, such studies provided higher doses of butyrate compared to the current study [72]. However, it is noteworthy that ENZ and BUT numerically increased the papillae width, and BUT also elevated the mitotic index, consistent with Sakata and Tamate [73] and Górka et al. [12].

In this study, ENZ and BUT did not significantly affect nutrient or total tract digestibility, similar to results by Rice et al. [67], who found no improvement in digestibility when supplementing sodium butyrate at 0.25 g/kg BW in heifers. Huhtanen et al. [74], using higher doses (400 g/d), observed positive effects on DM, NDF, and protein digestibility. A tendency for increased potential starch degradability was observed in animals supplemented with sodium butyrate, possibly due to subtle alterations in the ruminal microbial community, particularly the stimulation of amylolytic bacterial populations or enhanced starch fermentation activity. The lack of correspondence between greater potential starch degradability and ruminal propionate concentration may be due to several factors. First, the extent of starch fermentation may not have been sufficient to shift the SCFA molar proportions under the tested conditions. Second, part of the additional starch may have bypassed ruminal fermentation and been digested post-ruminally, as suggested by the unchanged SCFA profile. Previous studies have shown that butyrate can modulate microbial composition and fermentative patterns in the rumen, favoring bacteria involved in carbohydrate metabolism [13,49,75]. However, no significant differences were detected in the degradability of other nutrients or in overall nutrient digestibility with the inclusion of ENZ or BUT.

Despite no differences in nutrient or NDF digestibility among treatments, studies like Stahl et al. [76], using 0.75 g/kg BW, noted a tendency for increased digestibility in the control group compared to BUT. Given that most of the forage in this study’s diet was sugarcane bagasse, a highly lignified coproduct, the enzyme blend (glucanases and xylanases) likely had limited activity. In vitro, work by Ibáñez et al. [77] demonstrated that ENZ increased sugarcane bagasse digestibility and microbial protein yield, whereas Gómez-Vásquez et al. [78] indicated that a dosage of 15 to 30 g/d would be required to achieve a 65% improvement in bagasse digestibility—greater than the dosage used in the present study.

Finally, given that the central hypothesis was that increasing the butyrate concentration in the rumen would have beneficial effects on ruminal fermentation and metabolism, the results suggest that this increase was not effectively achieved, explaining the lack of significant effects. Consistent with previous studies (e.g., [14,79]), exogenous butyrate supplementation does not necessarily translate into greater measurable concentrations in the rumen because butyrate is rapidly absorbed across the ruminal epithelium and utilized by epithelial cells as an energy source. This likely explains why no significant differences in ruminal butyrate concentration were detected in our study, despite supplementation. Furthermore, this prompts a discussion on whether we should continue to pursue butyrate increases as the primary strategy for modulating the ruminal epithelium, as several studies have reported limited benefits [74,80,81]. This opens opportunities for new approaches that consider alternative metabolic pathways or combinations of additives with greater potential for efficacy.

## 5. Conclusions

The supplementation with sodium butyrate (BUT) and exogenous fibrolytic enzymes (ENZ) did not induce major changes in ruminal physiology or systemic metabolism in feedlot Angus cattle. Although some modest effects were observed, such as alterations in fermentative parameters, ruminal temperature, and protozoal population, these changes were neither statistically significant nor practically impactful. The hypothesis that increasing ruminal butyrate concentration or enzymatic activity would substantially modulate ruminal metabolism and promote ruminal epithelial development was not confirmed, possibly due to the doses used in this study. These findings indicate that BUT and ENZ supplementation exhibit preliminary promise; however, additional studies are required to elucidate their mechanisms and efficacy. Additionally, it is pertinent to note that future studies could quantify apoptosis in the ruminal epithelium to assess the impact of butyrate on this parameter. Associated with preliminary in vitro studies, future investigations should evaluate higher doses or combinations, quantify relevant metabolites and hormones to delineate the underlying physiological cascade, and include comprehensive performance assessments.

## Figures and Tables

**Table 1:** Composition and nutritional content of the experimental diets were identified as Adaptation Diet 1 (Adap1), Adaptation Diet 2 (Adap2), Adaptation Diet 3 (Adap3), and Finishing Diet.

Diets	Adap1 ^1^	Adap2 ^1^	Adap3 ^1^	Finishing ^2^
Ingredients, % of the dry matter				
Sugarcane Bagasse	12.00	12.00	12.00	12.00
Tifton Hay	18.00	13.00	8.00	3.00
Ground Corn	50.90	57.90	65.10	71.40
Soybean Meal	16.30	14.00	11.70	10.40
Urea	0.50	0.80	0.90	0.90
Mineral Supplement ^3^	2.30	2.30	2.30	2.30
Nutritional composition, % of the dry matter				
Crude Protein	15.50	15.40	14.50	14.30
Neutral Detergent Fiber	31.80	28.60	25.60	22.40
Physically Effective Neutral Detergent Fiber	23.00	20.00	16.00	13.00
Total Digestible Nutrients	74.00	76.00	78.00	80.00
Calcium	0.66	0.64	0.62	0.60
Phosphorus	0.44	0.44	0.43	0.44

^1^ Enzyme complex inclusion during adaptation was 0.01%, and sodium butyrate inclusion was 0.30%. ^2^ Enzyme complex inclusion during the finishing phase was 0.01%, and sodium butyrate inclusion was 0.1%. ^3^ Feed additives used in diets (enzyme complex and sodium butyrate) were added in the mineral supplement to act as a carrier and subsequently added into the diet at the time of feeding, directly in the feed bunk.

**Table 2:** Dry matter intake of rumen-cannulated Angus cattle fed diets with fibrolytic enzymes or butyrate.

	Treatments ^1^			SEM ^2^	*p*-Value
Items	CON	ENZ	BUT		Treatment
Dry matter intake, kg					
Adaptation	18.60	18.38	18.60	0.89	0.82
Finishing	20.68	20.19	21.23	1.56	0.78
Total Intake	19.60	19.25	18.86	1.16	0.76
% Body Weight	2.57	2.51	2.59	0.12	0.78

^1^ CON = Control; ENZ = Fibrolytic Enzyme (Rovabio); BUT = Sodium Butyrate (Adimix). ^2^ SEM = Standard Error of the Mean.

**Table 3:** Feeding behavior and particle size selectivity (PSPS) in rumen-cannulated Angus cattle fed with fibrolytic enzymes or butyrate.

	Treatments ^1^			SEM ^2^	*p*-Value
Items	CON	ENZ	BUT		Treatment
Sorting Index from PSPS					
Long	0.97 ^a^	0.76 ^b^	0.93 ^a^	0.04	<0.01
Medium	0.98	0.92	0.96	0.04	0.61
Short	1.01	1.04	1.04	0.01	0.18
Fine	0.98	0.95	0.93	0.03	0.16
Ingestive Behavior					
Idling Time, min/d	830.83	880.00	880.83	38.22	0.30
Ruminating Time, min/d	330.83	289.17	285.83	24.76	0.12
Feeding Time, min/d	231.67	214.17	227.50	23.09	0.53
DM Intake, kg	21.21	19.09	21.05	1.64	0.28
NDF Intake, kg	5.34	4.36	5.01	0.61	0.34
Meals per Day, n	15.67	17.33	17.00	1.00	0.48
Meal Duration, min	14.80	12.72	13.36	1.31	0.15
DM Intake per Meal, kg	1.39 ^a^	1.11 ^b^	1.25 ^ab^	0.11	0.06
Feeding Rate (DM), min/kg	10.72	11.45	10.89	0.84	0.21
Feeding Rate (NDF), min/kg of NDF	44.13	54.45	46.79	5.69	0.38
Ruminating Rate (DM), min/kg	15.97	15.28	14.21	1.63	0.31
Ruminating Rate (NDF), min/kg of NDF	66.02	70.50	64.21	8.61	0.83

^1^ CON = Control; ENZ = Fibrolytic Enzyme (Rovabio); BUT = Sodium Butyrate (Adimix). ^2^ SEM = Standard Error of the Mean. ^ab^ Within a row, means without a common superscript differ (*p* < 0.05).

**Table 4:** In situ ruminal degradability of nutrients in rumen-cannulated Angus cattle fed with fibrolytic enzymes or butyrate.

	Treatments ^1^			SEM ^2^	*p*-Value
Items, %	CON	ENZ	BUT		Treatment
Dry Matter Degradability					
Potential	82.7	78.88	81.34	2.2	0.43
At 2% passage rate	66.11	66.25	67.61	1.75	0.33
At 5% passage rate	54.2	54.77	55.02	1.99	0.85
At 8% passage rate	47.26	47.62	47.5	1.87	0.96
Neutral Detergent Fiber Degradability					
Potential	57.15	56.83	59.22	1.68	0.31
At 2% passage rate	52.33	52.32	52.67	1.16	0.96
At 5% passage rate	47.08	47.38	46.91	1.18	0.89
At 8% passage rate	43.28	43.76	42.86	1.26	0.61
Acid Detergent Fiber Degradability					
Potential	47.81	46.82	49.04	2.24	0.78
At 2% passage rate	45.64	45.07	45.40	1.27	0.94
At 5% passage rate	43.25	43.08	42.30	1.25	0.74
At 8% passage rate	41.45	41.58	40.59	1.41	0.86
Crude Protein Degradability					
Potential	86.41	86.22	88.57	2.05	0.14
At 2% passage rate	76.24	75.23	76.59	1.36	0.42
At 5% passage rate	65.86	64.32	64.85	0.91	0.42
At 8% passage rate	58.8	57.08	57.3	0.74	0.21
Starch Degradability					
Potential	98.12 ^b^	98.25 ^b^	99.54 ^a^	0.83	0.09
At 2% passage rate	83.8	84.09	84.35	1.24	0.77
At 5% passage rate	69.96	70.4	69.89	1.7	0.89
At 8% passage rate	60.93	61.44	60.62	1.81	0.81

^1^ CON = Control; ENZ = Fibrolytic Enzyme (Rovabio); BUT = Sodium Butyrate (Adimix). ^2^ SEM = Standard Error of the Mean. ^ab^ Within a row, means without a common superscript differ (*p* < 0.05).

**Table 5:** Total and nutrient digestibility in rumen-cannulated Angus cattle fed with fibrolytic enzymes or butyrate.

	Treatments ^1^			SEM ^2^	*p*-Value
Items	CON	ENZ	BUT		Treatment
Dry matter, %	94.56	94.23	94.36	0.39	0.69
Neutral detergent fiber, %	30.64	35.75	32.65	1.85	0.10
Acid detergent fiber, %	28.16	30.92	30.85	2.36	0.51
Crude protein, %	82.32	81.69	82.05	1.57	0.94
Starch, %	95.23	95.97	96.05	1.08	0.83
Fecal starch, %	10.92	7.65	8.90	1.98	0.51

^1^ CON = Control; ENZ = Fibrolytic Enzyme (Rovabio); BUT = Sodium Butyrate (Adimix). ^2^ SEM = Standard Error of the Mean.

**Table 6:** Rumen pH, temperature, and redox potential in rumen-cannulated Angus cattle fed with fibrolytic enzymes or butyrate.

	Treatments ^1^			SEM ^2^	*p*-Value
Items	CON	ENZ	BUT		Treatment
Maximum pH	6.70	6.45	6.46	0.18	0.38
Minimum pH	5.07	4.90	4.94	0.12	0.23
Mean pH	5.66	5.58	5.53	0.17	0.69
Time pH < 6.2, min	1149.17	1293.33	1230.83	122.53	0.45
Time pH < 5.6, min	770.83	777.50	850.83	207.05	0.92
Time pH < 5.2, min	435.00	314.17	571.67	189.74	0.51
Area under pH < 6.2	912.55	949.58	1031.97	176.55	0.88
Area under pH < 5.6	325.61	293.20	407.97	121.77	0.55
Area under pH < 5.2	81.98	71.21	116.00	47.83	0.68
Rumen temperature, °C	40.12 ^a^	39.63 ^b^	40.02 ^a^	0.37	0.04
Redox potential	−294.29	−288.85	−290.58	9.32	0.72

^1^ CON = Control; ENZ = Fibrolytic Enzyme (Rovabio); BUT = Sodium Butyrate (Adimix). ^2^ SEM = Standard Error of the Mean. ^ab^ Within a row, means without a common superscript differ (*p* < 0.05).

**Table 7:** Short-chain fatty acid profile in rumen-cannulated Angus cattle fed with fibrolytic enzymes or butyrate.

	Treatments ^1^			SEM ^2^	*p*-Value ^3^		
Items	CON	ENZ	BUT		Treatment	Hour	Trt*Hour
Acetate, %	61.05	60.85	61.45	3.63	0.86	0.23	0.96
Propionate, %	26.30	27.27	26.08	3.47	0.71	0.55	0.98
Butyrate, %	12.63	11.86	12.46	1.27	0.96	0.75	0.78
Total SCFAs, mmol/L ^4^	104.12	107.06	103.9	6.44	0.71	0.36	0.98
Acetate: Propionate ratio	2.49	2.51	2.48	0.29	0.99	0.89	0.88
NH_3_-N, mg/dL	3.80	3.61	3.62	0.67	0.74	<0.01	0.12
Lactate, mmol/L	0.99	0.63	1.09	0.29	0.28	0.04	0.10

^1^ CON = Control; ENZ = Fibrolytic Enzyme (Rovabio); BUT = Sodium Butyrate (Adimix). ^2^ SEM = Standard Error of the Mean. ^3^ Trt*Hour = Treatment*hour. ^4^ SCFAs = Short-Chain Fatty Acids.

**Table 8:** Differential count of ciliate protozoa (10^3^/mL) in rumen-cannulated Angus cattle fed with fibrolytic enzymes or butyrate.

	Treatments ^1^			SEM ^2^	*p*-Value
Items	CON	ENZ	BUT		Treatment
Absolute values					
Isotricha	21.20	22.20	15.40	3.68	0.12
Dasytricha	23.00	25.60	19.20	3.83	0.24
Entodinium	108.00	140.80	98.60	25.96	0.15
Diplodinium	10.20 ^a^	7.40 ^b^	12.20 ^a^	2.35	<0.01
Total	162.40	196.00	145.40	32.65	0.23
Percentage of the total count					
% Isotricha	13.98 ^a^	11.21 ^b^	10.76 ^b^	1.01	0.02
% Dasytricha	15.30	14.15	13.46	1.63	0.69
% Entodinium	64.06	71.08	66.15	2.94	0.16
% Diplodinium	6.66 ^ab^	3.56 ^b^	9.64 ^a^	1.82	0.03

^1^ CON = Control; ENZ = Fibrolytic Enzyme (Rovabio); BUT = Sodium Butyrate (Adimix). ^2^ SEM = Standard Error of the Mean. ^ab^ Within a row, means without a common superscript differ (*p* < 0.05).

**Table 9:** Body weight, rumen content fractions, and disappearance rate in rumen-cannulated Angus cattle fed with fibrolytic enzymes or butyrate.

	Treatments ^1^			SEM ^2^	*p*-Value
Items	CON	ENZ	BUT		Treatment
Final body weight, kg	776.82	764.67	781.43	10.89	0.33
Liquid mass, kg	61.04	55.86	59.21	5.30	0.51
Solid mass, kg	13.86	12.47	13.48	1.05	0.59
Total rumen mass, kg	74.90	68.33	72.68	5.22	0.52
Liquid mass, % of body weight	7.87	7.25	7.64	0.52	0.52
Solid mass, % of body weight	1.82	1.63	1.75	0.19	0.61
Total rumen mass, % of body weight	9.69	8.87	9.39	0.60	0.53
Dry matter–rumen content	18.84	18.26	18.85	2.02	0.79
Disappearance rate, %/h	6.36 ^b^	8.61 ^a^	7.12 ^ab^	1.11	0.05
Disappearance rate, kg/h	0.86 ^b^	0.96 ^a^	0.93 ^ab^	0.07	0.09

^1^ CON = Control; ENZ = Fibrolytic Enzyme (Rovabio); BUT = Sodium Butyrate (Adimix). ^2^ SEM = Standard Error of the Mean. ^ab^ Within a row, means without a common superscript differ (*p* < 0.05).

**Table 10:** Blood hormones and inflammation-related proteins in rumen-cannulated Angus cattle fed with fibrolytic enzymes or butyrate.

	Treatments ^1^			SEM ^2^	*p*-Value
Items	CON	ENZ	BUT		Treatment
C-reactive protein, mg/L	6.52 ^b^	6.43 ^b^	7.68 ^a^	0.49	0.09
Lipopolysaccharide-binding protein, ng/mL	376.37 ^a^	328.70 ^b^	343.10 ^a,b^	13.09	0.02
Immunoglobulin G, µg/mL	89.15	85.97	109.01	12.61	0.17
Immunoglobulin A, µg/mL	12.63	13.67	14.25	2.93	0.51
Haptoglobin, µg/mL	143.36	131.49	148.69	69.98	0.25
Ceruloplasmin, IU/L	32.01	33.20	35.26	5.79	0.50
Serum amyloid A, µg/mL	8.29	7.66	8.13	0.39	0.34
Insulin, mIU/L	24.56	21.63	30.89	2.85	0.18
Non-esterified fatty acids, µmol/L	126.86	122.12	125.16	33.48	0.99
Glucose, mg/dL	222.25	245.23	200.69	116.69	0.72

^1^ CON = Control; ENZ = Fibrolytic Enzyme (Rovabio); BUT = Sodium Butyrate (Adimix). ^2^ SEM = Standard Error of the Mean. ^a,b^ Within a row, means without a common superscript differ (*p* < 0.05).

**Table 11:** Histological variables of rumen papillae in rumen-cannulated Angus cattle fed with fibrolytic enzymes or butyrate.

	Treatments ^1^			SEM ^2^	*p*-Value
Items	CON	ENZ	BUT		Treatment
Height, mm	7.84	8.28	8.14	0.41	0.73
Width, mm	2.44	2.69	2.68	0.14	0.21
Papilla area, mm^2^	19.35	22.37	21.88	1.76	0.44
Keratin layer thickness, µm	20.77	20.02	20.79	1.02	0.75
Mitotic index, count, n	230.17	242.17	271.83	13.04	0.23
Mitotic index, percentage, %	12.11	13.01	13.59	0.65	0.23

^1^ CON = Control; ENZ = Fibrolytic Enzyme (Rovabio); BUT = Sodium Butyrate (Adimix). ^2^ SEM = Standard Error of the Mean.

## Data Availability

Data will become available upon request to the corresponding author.
